# Geriatric nutritional risk index predicts all-cause mortality in patients with heart failure: A systematic review and meta-analysis

**DOI:** 10.6061/clinics/2021/e2258

**Published:** 2021-03-15

**Authors:** Chao-hui Dong, Si-yu Chen, Hong-lian Zeng, Bo Yang, Jia Pan

**Affiliations:** I Department of Health Management Center, Affiliated Hospital of Chengdu University, Jinniu district, Chengdu City, Sichuan Province, China; II Department of Vascular Surgery, Sichuan Academy of Medical Sciences, Sichuan Provincial People's Hospital, Qingyang district, Chengdu City, Sichuan Province, China

**Keywords:** Geriatric Nutritional Risk Index, Heart Failure, All-Cause Mortality

## Abstract

**OBJECTIVES:**

Geriatric nutritional risk index (GNRI) might predict the all-cause mortality in patients with heart failure (HF). We performed a meta-analysis to evaluate the correlation between GNRI and all-cause mortality in patients with HF.

**METHODS:**

We searched the PubMed, Medline, Cochrane Library, and Embase databases for clinical trials investigating the association between GNRI and all-cause mortality in patients with HF, having the primary endpoint as all-cause mortality.

**RESULTS:**

In total, nine studies involving 7,659 subjects were included in the systematic review and meta-analysis. The results indicated that major risk and moderate risk GNRI (GNRI<92) was associated with an increased risk of all-cause mortality in elderly patients with HF (hazard ratios [HR] 1.59, 95% confidence intervals [CI] 1.37-1.85). Low risk GNRI (GNRI<98) group predicted all-cause mortality in elderly HF patients (HR 1.56, 95%CI 1.12-2.18) when compared with the high GNRI value group. A subgroup analysis indicated that the relationship between GNRI and HF might differ based on the subtype of heart failure.

**CONCLUSIONS:**

GNRI is a simple and well-established nutritional assessment tool to predict all-cause mortality in patients with HF.

## INTRODUCTION

Heart failure (HF) is associated with high morbidity and mortality. Approximately 1-2% of adults have HF, and the incidence increases to 10% among people aged above 70 years (1-4). The all-cause mortality rate has been reported to be 5.0-30.0% in 12 months, and the one-year hospitalization rates are 18.9-65.0% (5-7). Some studies indicated that malnutrition was common in patients with HF, and it might be useful in predicting the all-cause mortality, risk of cardiovascular (CV) events, and hospitalization for HF (11-16). Among malnutrition scores, the prognostic nutrition status (PNI), controlling nutrition status (CONUT), and geriatric nutritional risk index (GNRI) have been studied, and the GNRI is reported to be very important in elderly patients. The GNRI is calculated based on the serum albumin and body mass index (BMI). It could be a simple tool in predicting the prognosis for various cancers (8,9), such as large B-cell lymphoma, esophageal squamous cell carcinoma, and others. Some studies indicated that it could also be a simple and well-established nutritional assessment tool and a significant prognostic factor for HF. However, there have been no systematic reviews and meta-analyses on the association between GNRI and HF.

The objective of our meta-analysis was to investigate the relationship between GNRI and all-cause mortality among patients with HF.

## MATERIALS AND METHODS

### Search strategy

We searched for articles in PubMed, Medline, Embase, and the Cochrane Library published up to November 15, 2019, without language restrictions. The following MESH terms or text words were used for our search: HF, GNRI, malnutrition, and mortality. Completed trials among human beings whose abstracts or full texts were available were selected.

### Geriatric nutritional risk index

The GNRI was calculated from the serum albumin and BMI:

GNRI=14.89* serum albumin (g/dl) +41.7*BMI/22 kg/m^2^

The cut-off values of GNRI were based on the report by Bouillanne et al. (10). They defined four grades of nutrition-related risk of morbidity and mortality for elderly patients: major risk (GNRI<82), moderate risk (82≤GNRI<92), low risk (92≤GNRI<98), and no risk (GNRI>98). We defined two groups of nutrition-related risk in our meta-analysis as follows: a low GNRI group (GNRI<92) with a risk of malnutrition-related morbidity and mortality and a high GNRI (GNRI≥92) group with low or no risk of malnutrition- related morbidity and mortality.

### Inclusion and exclusion criteria

Two investigators independently reviewed the title and abstracts of all potentially eligible studies reporting the effect of GNRI on all-cause mortality in patients. Two researchers independently extracted the data using standardized data extraction forms and the defined eligibility criteria. Disagreements were solved by consensus. To ensure the reliability of the studies, the following criteria were used for the selection: a) Adults patients with HF, b) GNRI available and associated with all-cause mortality, c) cut-off GNRI value of 92, The following studies were excluded: a) overlapping or duplicate reports, b) Non-human experiments, c) The cut-off value not meeting our defined criteria.

### Data extraction and quality assessment

After removing all duplicate citations, two researchers extracted all the outcome data independently. The following data were extracted: publication characteristics, study regions, follow-up duration, BMI, GNRI, serum albumin, and all-cause mortality/CV events. The quality of the studies selected was scored by two reviewers using Newcastle-Ottawa Scale (NOS). We considered studies as high quality if they had a score of more than six.

### Statistical analysis

All data were analyzed using STATA statistical software (version 14.0). Results are presented as mean±x when normally distributed and as median and interquartile range otherwise. Heterogeneity between the studies was evaluating the Cochrane’s Q and I2 tests. A fixed-effect model was used in the absence of significant heterogeneity (I2<50%), else the random effect model was used. Publication bias in studies with different samples size was assessed by Egger’s funnel plots. We considered two-sided probability values of <0.05 as statistically significant.

## RESULTS

### Literature search and included studies

**Figure 1:**
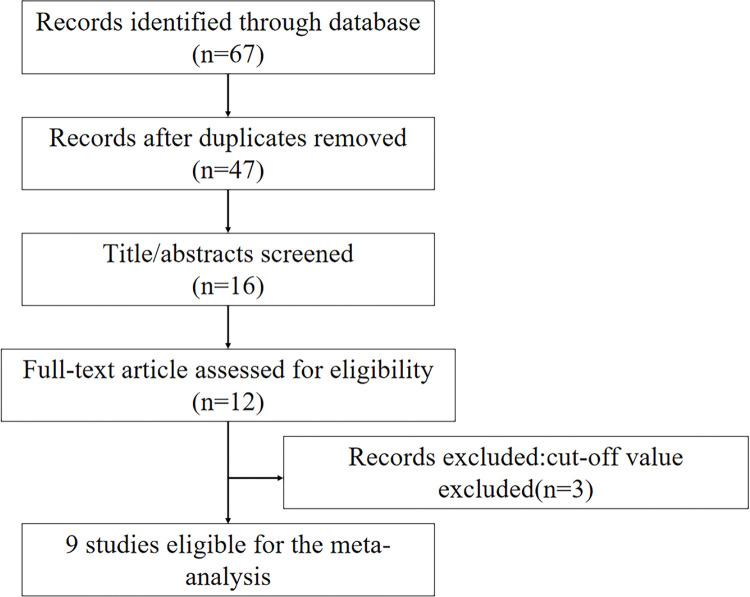
Flowchart of the selection of published studies selected for the meta-analysis.

Initially, a total of 47 articles were selected from the primary search in the major databases. After screening the title and abstracts, 31 articles were excluded. Finally, nine studies published from 2013 to 2019 were selected (11-19). The flow diagram of the study selection is shown in Figure 1. A total of 7,659 patients were enrolled, and the characteristics of the included studies are listed in Table 1. All of them were observational studies, and six studies were conducted in Japan. All the studies reported all-cause mortality, while four studies also reported CV events. The mean follow-up duration was 2.8±1.0 years (range 189 days to 1,573 days). The mean age of the enrolled patients was 74.7±5.1 years, and 37.9% of them were female. Three studies included HF patients with preserved ejection fraction (11-12,19), while two studies included patients with acute HF (17-18), four studies had a mixed cohort (13-16). The hazard ratios (HRs) and corresponding 95% confidence intervals (CIs) were selected from each study. According to the Newcastle-OttawaScale scores (NOS), all cohort studies were of high quality and had scores of six or more.

### GNRI and all-cause mortality in HF

**Figure 2:**
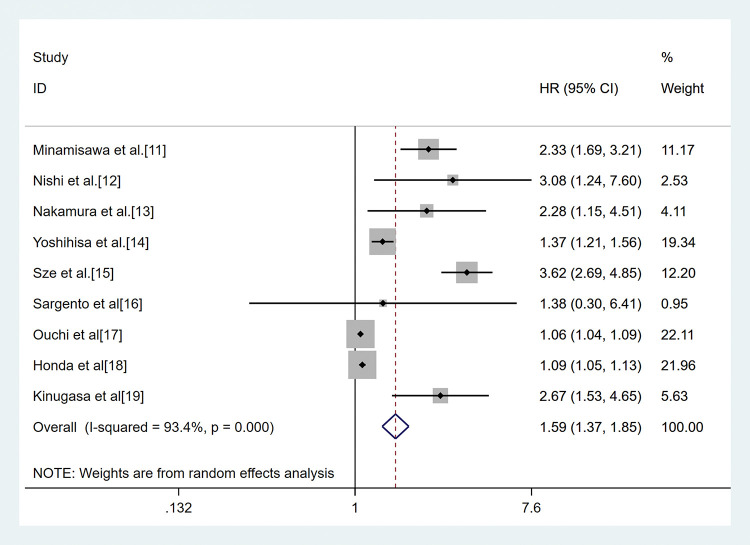
Result of the meta-analysis of HR values for all-cause mortality (the cut-off value of GNRI was 92). Each square denotes the HR for each trial compared with the corresponding 95% confidence intervals. The size of the square is directly proportional to the amount of information contributed by the trial. The diamonds represent the overall outcome of HR and 95%CI.

**Figure 3:**
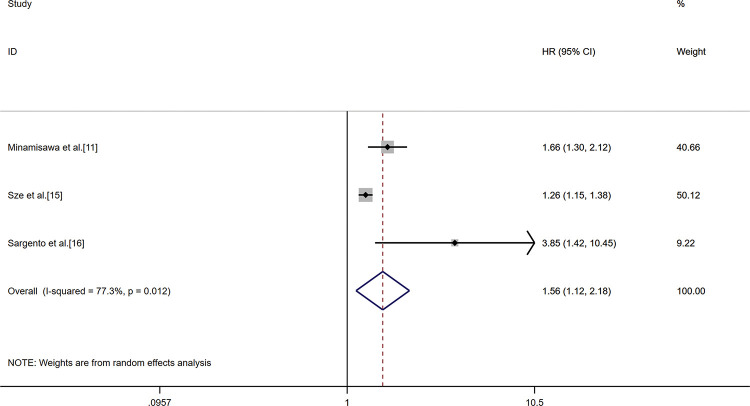
Result of the meta-analysis of HR values for all-cause mortality (the cut-off value of GNRI was 98). Each square denotes the HR for each trial compared with the corresponding 95% confidence intervals. The size of the square is directly proportional to the amount of information contributed by the trial. The diamonds represent the overall outcome of HR and 95%CI.

The combined analysis of nine cohorts, including 7,659 patients, showed the association between GNRI and all-cause mortality. Patients were divided into two malnutrition-related risk groups: moderate to serve risk (GNRI<92), low or no risk (GNRI≥92). The pooled outcome for the low GNRI group showed a 1.59 (95%CI 1.37-1.85) times higher risk compared with the high GNRI group (*p*<0.01, random effect, Figure 2) for all-cause mortality. Three studies had a GNRI cut-off value of 98 (11,15-16). The analysis indicated that the low GNRI group had higher all-cause mortality in HF (HR 1.56, 95%CI 1.12-2.18, (*p*<0.01, random effect) compared to the high GNRI group (Figure 3).

### Subgroup analysis of GNRI and all-cause mortality

Due to extreme heterogeneity between the studies (I^2^=93.7%, *p*<0.01), we conducted four subgroup analyses based on the study region (western countries, Eastern countries), sample size (>500 and ≤500), age (>75 years and ≤75 years), follow-up duration (>2 years and ≤2 years), and type of heart failure [heart failure with preserved ejection fraction (HFpEF), Acute heart failure, Mixed]. When grouped by study region, GNRI predicted the all-cause mortality in Western countries (HR 2.78, 95%CI 1.86-4.17, random effect) (11,15-16) as well as Eastern countries (HR 1.21, 95%CI 1.09-1.34, random effect) (12-14,17-19). On comparison by sample size, similar results were seen in groups with large sample (HR 2.24, 95%CI 1.20-2.18, random effect) (11,14-15) and small sample size (HR 1.13, 95%CI 1.03-1.24, random effect) (12-13,16,18-19). The prognostic role of GNRI in predicting all-cause mortality was seen in subjects >75 years old (HR 1.91, 95%CI 1.07-3.38, random effect) (12-13,15,17-18) and ≤75 years old (HR 1.82, 95%CI 1.21-2.73, random effect) (11,14-15,17-18). GNRI predicted all-cause mortality in the groups with short duration (HR 1.09, 95%CI 1.02-1.17, random effect) (12-13,15,17-18) as well as long duration of follow-up (HR 2.23, 95%CI 1.35-3.68, random effect) (11,14-16,19). Three studies enrolled patients with HFpEF (11-12,19), while two studies included patients of acute heart failure (17-18), four studies included a mixed cohort. GNRI predicted all-cause mortality in the HFpEF group (HR 2.46, 95%CI 1.88-3.21, random effect), acute heart failure group (HR 1.07, 95%CI 1.05-1.09, random effect), and the mixed group (HR 2.10, 95%CI 1.07-4.12, random effect). The results are listed in Table 2.

### Sensitivity analysis and publication bias

**Figure 4:**
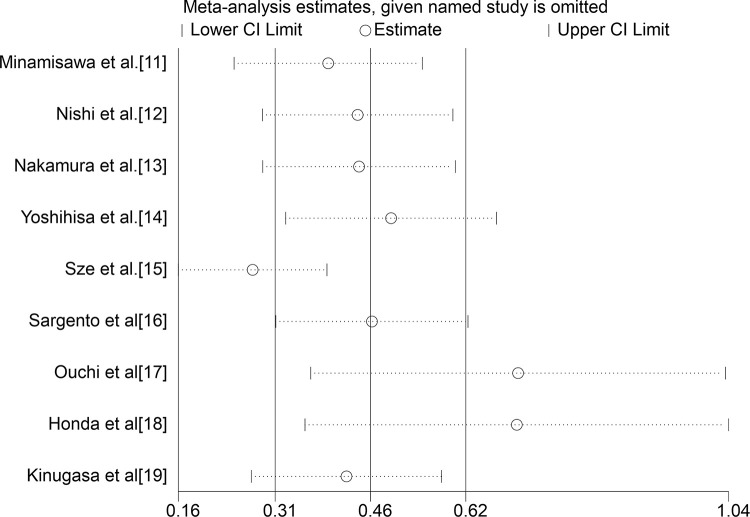
Result of the sensitivity analysis. The middle vertical line indicates the combined OR, and the two vertical lines represent the 95% CI values. Every round hollow indicates the pooled OR when the left study was omitted in a meta- analysis with a random model.

Sensitivity analysis indicated that some included studies led to heterogeneity in the meta-analysis (Figure 4). The subgroup analysis indicated that the type of HF might be the main cause of heterogeneity, and further studies should focus on the predictive role of GNRI by the subtype of HF. There was some publication bias seen on Egger’s funnel plot, which might have been caused due to the small number of studies included.

## DISCUSSION

HF is a growing health problem and is associated with high mortality. In patients with HF, malnutrition is common due to the change in systemic metabolism, the increase of body consumption and the dysfunction of gastrointestinal. A previous study indicated that approximately one-third of the patients with HF had accompanying malnutrition (11) and that malnutrition was associated with an increased risk for CV events, hospitalization due to HF, and all-cause death.

The Geriatric Nutritional Risk Index (GNRI) is an-elderly-specific index that has been proposed to assess the nutrition-related risk of morbidity and mortality for elderly patients.Some studies demonstrated that low GNRI was associated with an increased risk for cancer and other diseases (8,20). Our meta-analysis confirmed that GNRI could be a simple and feasible tool to assess nutrition in patients with HF.

The present study indicated that GNRI was associated with an increased risk for all-cause mortality in patients with HF. Patients in our study were divided into two groups, low GNRI (<92) indicating moderate to serve malnutrition, and high GNRI (≥92) indicating low or no risk of malnutrition. Our meta-analysis indicates that low GNRI predicted all-cause mortality in patients with HF (*p*<0.05). A further analysis involving 5206 subjects demonstrated that even a low risk of malnutrition (<98) was associated with an increased risk of all-cause mortality in patients with HF, which was in line with previous studies. Mastatoshi et al. also reported that low GNRI was strongly associated with increased CV events and re-hospitalization due to HF.

In our subgroup analysis, we grouped patients by region, age, sample size, follow-up duration, and subtype of HF. The analysis of the correlation between GNRI and clinical features demonstrated the prognostic role of GNRI in predicting mortality was higher in studies in the Western countries, those with large sample size, and having a long-term follow-up. One study enrolled extremely old patients (>80 years old), while patients in the other studies were elderly (≥65 years old). The result demonstrated that an association between low GNRI and poor prognosis in patients with HF was seen at all ages. In the analysis by subtype of HF, the significance of GNRI in predicting all-cause mortality in acute HF was low, while it was high in chronic HF. Thus, low GNRI might be better in predicting all-cause mortality in chronic heart failure than acute heart failure, especially in HFpEF.

There was some heterogeneity in our study; we conducted some subgroup and sensitivity analyses to detect the origin of the heterogeneity. The subgroup analysis indicated the type of HF as the main cause of heterogeneity. The included studies comprised patients with acute heart failure, HFpEF, HFrEF, and mixed type of heart failure, which might lead to some heterogeneity. However, the limited number of studies precluded a more detailed analysis of classification.

GNRI was calculated from serum albumin and BMI, and was a more useful parameter for predicting mortality than BMI and serum albumin in HF. Seven of the included studies reported BMI, of which the mean BMI was above 28 kg/m^2^ in one study, while it was below 28 kg/m^2^ in others. GNRI might underestimate malnutrition in overweight patients. Moreover, different nutrition-related risk results might be seen with different assessment methods, such as CONUT or PNI. Hence, future studies should focus on a better method to estimate the nutrition in HF patients.

To the best of our knowledge, this is the first meta-analysis that analyzed the association between GNRI and all-cause mortality in patients with HF. However, there are several limitations of the present systematic review and meta-analysis. First, some of the included studies had a small sample size in the low GNRI group, which could have affected the results. Second, studies that lacked information concerning the GNRI and mortality were excluded from the analyses. Third, the number of women enrolled was lower than men, which might have influenced the results. The population in studies from Eastern countries was entirely from Japan, which might have influenced some results. Besides, all the included studies were observational studies, which can influence the accuracy of the study. Despite these limitations, GNRI could be an effective marker in predicting the risk of mortality in HF.

## CONCLUSIONS

In elderly patients with HF, GNRI is a simple and well-established nutritional assessment tool that can help in predicting all-cause mortality. More studies should focus on GNRI and the subtype of HF and suggest methods to improve malnutrition in patients with HF.

## AUTHOR CONTRIBUTIONS

Dong CH and Chen SY designed and conceived this study. Dong CH and Chen SY completed data collection and manuscript writing. Pan J participated in data analysis and manuscript preparation. Zeng HL and Yang B provided important suggestions for article revision. Chen SY approved the final version of the manuscript.

## Figures and Tables

**Table 1:** Main characteristics of the included studies.

Study	Heart failure type	Number of patients	Age (year, mean±s)	Female (n,%)	Follow-up	Country	All-cause mortality/CV events	BMI kg/m^2^	Serum albumin (g/l)	NOS
Minamisawa et al. (11)	HFpEF	1677	72.4 (64.1, 79.5)	516 (48.5)	2.9 years	USA, Canada, Brazil, Argentina	All-cause mortality, CV events	32.4 (27.8,37.8)	4.1 (3.9,4.3)	8
Nishi et al. (12)	HFpEF	110	78.5±7.2	51 (46.4)	503 days	Japan	All-cause mortality	23.1±4.1	3.6 (3.2,3.9)	7
Nakamura et al. (13)	HF	213	87.2±4.9	120 (56.3)	540 days	Japan	All-cause mortality	low 18.2±2.3 high 23.0±3.0	low 3.2±0.4 high 3.7±0.3	7
Yoshihisa et al. (14)	HF	1274	66.5	482 (37.8)	1146 days	Japan	All-cause mortality	NA	NA	7
Sze et al. (15)	HF	3386	75.0 (67.0, 81)	1320 (39.0)	1573 days	UK	All-cause mortality	NA	NA	7
Sargento et al. (16)	HFrEF	143	75.4±6.5	43 (30.1)	3 years	Portugal	All-cause mortality	28.8±7.9	4.1±0.5	7
Ouchi et al. (17)	Acute HF	214	73.0 (64.0, 82.0)	99 (46.3)	22.6±14.2 months	Japan	All-cause mortality	22.9 (20.4-25.7)	3.4 (3.0,3.6)	7
Honda et al. (18)	Acute HF	490	79.0±7.0	203 (41.0)	189 days	Japan	All-cause mortality, CV events	22.6±3.7	3.8 (3.4,4.0)	7
Kinugasa et al. (19)	HFpEF	152	77.0±11.0	70 (46.1)	2.1 years	Japan	All-cause mortality, CV events	21.7±4.0	3.5±0.5	7

HFpEF heart failure with preserved ejection fraction; CV cardiovascular; BMI body mass index; NOS Newcastle-Ottawa Scale.

**Table 2:** Results of the subgroup analysis on prognostic significance of GRNI in HF.

Analysis	Categories	No of Studies (n)	Patients (n)	Model	HR (95%CI)	Z	*p*	I^2^ (%)	*p_h_*
Overall		9	7659	Random	1.59 (1.37, 1.85)	5.96	0.00	93.40	0.00
Study region	Western country	3	5206	Random	2.78 (1.86, 4.17)	4.95	0.00	58.70	0.08
	Eastern country	6	2453	Random	1.21 (1.09, 1.34)	3.58	0.00	85.80	0.00
Sample size	>500	3	6337	Random	2.24 (1.20, 4.18)	2.52	0.01	95.00	0.00
	≤500	6	1332	Random	1.13 (1.03, 1.24)	2.57	0.01	76.30	0.00
Age	>75 years	4	1108	Random	1.91 (1.07, 3.38)	2.21	0.02	79.50	0.00
	≤75 years	5	6551	Random	1.82 (1.21, 2.73)	2.89	0.00	97.00	0.00
Follow-up duration	>2 years	5	6632	Random	2.23 (1.35, 3.68)	3.14	0.00	90.60	0.00
	≤2 years	4	1027	Random	1.09 (1.02, 1.17)	2.48	0.01	72.10	0.01
HF type	Acute HF	2	704	Random	1.07 (1.05, 1.09)	6.24	0.00	0.00	0.36
	HFpEF	3	1939	Random	2.46 (1.88, 3.21)	6.61	0.00	0.00	0.81
	Mixed	4	5106	Random	2.10 (1.07, 4.12)	2.15	0.03	91.70	0.00

GRNI geriatric nutritional risk index; HF heart failure; HFpEF heart failure with preserved ejection fraction; HR hazard ratios; CI confidence interval; *p_h_ p* value of heterogeneity.
